# Disseminated tuberculosis with Poncet's disease mimicking childhood-onset systemic lupus erythematosus: a unique case report

**DOI:** 10.1590/S1678-9946202668043

**Published:** 2026-07-20

**Authors:** Matheus Santos França, Fernanda Lima Gomes, Marina de Azambuja Nogueira, Maria Fernanda Bádue Pereira, Heloisa Helena de Sousa Marques, Katia Tomie Kozu, Lucia Maria Arruda Campos, Clovis Artur Silva

**Affiliations:** 1 Universidade de São Paulo, Faculdade de Medicina, Hospital das Clínicas, Instituto da Criança e do Adolescente, Unidade de Reumatologia Pediátrica, São Paulo, São Paulo, Brazil; 2 Universidade de São Paulo, Faculdade de Medicina, Hospital das Clínicas, Instituto da Criança e do Adolescente, Unidade de Infectologia Pediátrica, São Paulo, São Paulo, Brazil

**Keywords:** Poncet's disease, Tuberculosis, Childhood-onset systemic lupus erythematosus

## Abstract

Disseminated tuberculosis in children may present with systemic and immunologic features that overlap with autoimmune diseases, complicating diagnosis and treatment. We report the first pediatric case of microbiologically confirmed disseminated tuberculosis involving multiple sites associated with Poncet's disease, mimicking childhood-onset systemic lupus erythematosus (cSLE). A previously healthy five-year-old boy presented with a four-month history of persistent fever, weight loss, and symmetrical oligoarthritis involving both knees and wrists, resulting in loss of mobility. Laboratory tests revealed severe non-hemolytic anemia, leukopenia, elevated inflammatory markers, hypoalbuminemia, hematuria, positive antinuclear antibodies, low complement levels, and lupus anticoagulant positivity. Chest imaging showed bilateral pleural effusion, pulmonary opacities, and lymphadenopathy, fulfilling the Systemic Lupus International Collaborating Clinics (SLICC) classification criteria for cSLE. Despite initially negative tuberculin skin test and gastric lavage analysis, lymph node biopsy confirmed *Mycobacterium tuberculosis* by histology, molecular assay, and culture. Subsequent urine and gastric samples were also positive. The patient had received BCG vaccination at birth. He was treated with rifampicin, isoniazid, pyrazinamide, and pyridoxine, with rapid clinical improvement, complete resolution of arthritis, and full recovery of mobility without sequelae in one month. No immunosuppressive therapy was administered. This case reinforces the diagnostic complexity of distinguishing tuberculosis from childhood-onset systemic autoimmune rheumatic diseases, particularly in endemic regions. To our knowledge, this is the first reported pediatric case of disseminated tuberculosis with Poncet's disease mimicking cSLE. Recognizing this specific condition is crucial to prevent misdiagnosis and unnecessary immunosuppression, while ensuring timely treatment of the underlying infection.

## INTRODUCTION

Tuberculosis (TB) remains a major cause of morbidity and mortality in children. In endemic regions, its diverse clinical manifestations may mimic autoimmune rheumatic and inflammatory diseases, posing significant diagnostic challenges. Furthermore, TB may coexist with autoimmune rheumatic conditions, further complicating diagnosis and treatment, particularly when distinguishing infectious manifestations from immune-mediated disease activity.

Poncet's disease (PD) is a rare form of reactive arthritis that occurs during active TB infection without direct bacillary invasion of the joints. It is typically non-erosive and non-deforming and resolves completely with antitubercular therapy. Due to overlapping features with systemic autoimmune conditions, such as childhood-onset systemic lupus erythematosus (cSLE), diagnosis may be challenging.

We report the first known pediatric case of disseminated TB with microbiological confirmation from multiple sites associated with Poncet's disease, mimicking cSLE and fulfilling the Systemic Lupus International Collaborating Clinics (SLICC) classification criteria for this pediatric autoimmune rheumatic condition.

### Ethics

This study was approved by the Institutional Review Board of the Instituto da Crianca e do Adolescente, Hospital das Clinicas, Faculdade de Medicina, Universidade de Sao Paulo (CAAE Nº 94892725.8.0000.0068, approval Nº 8.152.058). Written informed consent for publication was obtained from the patient's legal guardians.

## CASE REPORT

A previously healthy five-year-old boy was admitted with a four-month history of persistent daily fever, with up to six spikes per day and maximum temperatures reaching 40 °C, alternating with nocturnal hypothermia to 35 °C. The febrile illness was accompanied by progressive weight loss, totaling approximately 18% of his baseline body weight. Over time, he developed symmetrical oligoarthritis involving both knees and wrists, leading to a semiflexed position of the lower limbs due to severe pain and progressing to loss of mobility by the time of admission. Chest computed tomography revealed bilateral pleural effusion, more pronounced on the right side, along with ground-glass pulmonary opacities and bilateral hilar and right axillary lymphadenopathy. He had received Bacillus Calmette-Guerin (BCG) vaccination at birth, in accordance with Brazil's national immunization program.

Laboratory evaluation showed normocytic, normochromic anemia (Hb 5.3 g/dL; MCV 76 fL; MCH 23 pg; RDW 15.2%), requiring red blood cell transfusion, with normal direct Coombs test. Marked leukopenia (2,530 cells/mm^3^) and lymphopenia (590 cells/mm^3^) were observed, while platelet count remained within the normal range. Inflammatory markers were elevated (CRP 90 mg/L; ESR 97 mm/h), accompanied by hypoalbuminemia (2.8 g/dL) and persistent microscopic hematuria. Serum calcium was elevated (11.5 mg/dL; reference range: 8.8–10.8 mg/dL), with suppressed parathyroid hormone (PTH) levels (<6 pg/mL; reference range: 28–129 pg/mL).

Knee ultrasound showed moderate joint effusion with synovial proliferation. Immunologic evaluation revealed positive antinuclear antibodies (ANA) with mixed patterns: nuclear fine speckled (AC-4) at 1:1280 and cytoplasmic fibrillar linear (AC-15) at 1:160. Lupus anticoagulant was positive, and total complement activity (CH50) was reduced to 18.94 U/mL (reference range: 42–95 U/mL). Anti-Sm, anti-SSA/Ro, anti-SSB/La, anti-RNP, and anti-dsDNA autoantibodies were negative.

Initial evaluation for TB showed a non-reactive tuberculin skin test (TST). Molecular testing and smear microscopy were negative in pleural fluid and in three gastric lavage samples. Biopsy of the right axillary lymph node revealed caseating granulomas and tested positive for *Mycobacterium tuberculosis* by both rapid molecular assay (Xpert MTB/RIF Ultra^®^, Cepheid) and culture. Subsequent urine samples were also positive for *Mycobacterium tuberculosis* by molecular assay. After 20 days, culture of the initial gastric lavage sample also yielded *Mycobacterium tuberculosis*.

Human immunodeficiency virus (HIV) infection was excluded by serology. Anti-tuberculosis treatment with rifampicin, isoniazid, pyrazinamide, and pyridoxine was initiated. Within one week, the patient showed clinical improvement, including resolution of fever and progressive recovery of overall condition. After one month, he regained independent lower limb mobility, with complete resolution of joint symptoms and no sequelae. During anti-tuberculosis treatment, laboratory abnormalities also improved: hemoglobin increased to 11.6 g/dL, total leukocyte count to 3,170 cells/mm^3^ (including an absolute lymphocyte count of 1,100 cells/mm^3^), and microscopic hematuria resolved. He remains under multidisciplinary follow-up with pediatric infectious diseases, rheumatology, and immunology units of our tertiary care hospital.

## DISCUSSION

To our knowledge, this is the first pediatric case of disseminated TB with microbiological confirmation from multiple sites associated with Poncet's disease fulfilling classification criteria for cSLE. This case highlights the complexity of distinguishing TB from autoimmune rheumatic conditions in children, particularly in endemic regions, where infectious and immune-mediated diseases often overlap in clinical and laboratory features.

Brazil is currently, and has historically been, among the 30 high-burden countries for TB, as classified by the World Health Organization. In 2024, 4.1% (n=3,468) of the 84,308 newly reported cases occurred in children and adolescents under 15 years of age^1,2^. However, the true incidence of pediatric TB is likely underestimated, as the disease may be underdiagnosed in this population, even in high-burden settings^3^.

Several factors contribute to this diagnostic gap, including the paucibacillary nature of pediatric TB, challenges in sample collection, limited sensitivity of available diagnostic tests, and, at times, low clinical suspicion. Although obtaining appropriate specimens from pediatric patients can be difficult, evidence suggests that testing multiple samples in cases of intrathoracic TB may increase cumulative microbiological yield by up to 33%, as observed in the present case^4,5^. This approach may also be beneficial for extrapulmonary and disseminated disease. In pulmonary TB, the use of alternative specimens, such as stools or nasopharyngeal aspirate, has demonstrated good diagnostic sensitivity^4,6^. When other organs or systems are affected, biopsy or on-site sample collection—such as lymph node aspirates or urine samples, in this case—may further improve diagnostic yield^6^.

The TST is an established method for detecting immunologic sensitization to *Mycobacterium tuberculosis* antigens. However, it may be nonreactive in approximately 10%–20% of pediatric tuberculosis disease^7^. Therefore, this case underscores the importance of pursuing microbiological confirmation to ensure accurate diagnosis and appropriate clinical management, even in children with negative TST results. Achieving this often requires maintaining a high index of suspicion and employing multiple specimen types and analytic techniques to enhance sensitivity and overall diagnostic accuracy.

BCG vaccination administered soon after birth is a key strategy in many countries, including Brazil, to prevent disseminated TB in children under five years of age^8^. In a meta-analysis, BCG effectiveness in this age group was estimated at 0.63 (95% CI 0.49–0.81)^9^. Despite this protective effect, TB may still progress to lymphohematogenous dissemination and subsequently to extrapulmonary disease, especially in young children, as observed in this case. Unlike adults, in whom pleural TB is more common, lymph node tuberculosis is the most frequent form of extrapulmonary TB in children. Disseminated TB, defined as involvement of two or more organ systems, accounts for approximately 5% of cases^10^, while the distribution of other extrapulmonary sites in the pediatric population varies across studies^7,10^.

Furthermore, musculoskeletal TB accounts for approximately 10% of extrapulmonary TB cases, with spinal involvement comprising nearly half of these. Among nonvertebral presentations, the hips, knees, and para-articular regions are most frequently affected^11^. Typically, TB arthritis presents as a chronic monoarthritis with potential joint destruction and histologic evidence of granulomatous inflammation. In contrast, our patient developed a symmetrical oligoarthritis with rapid clinical resolution following antitubercular therapy—a pattern more consistent with a reactive process than with true infectious arthritis.

The association between TB and polyarthritis has been recognized since the 19^th^ century. In 1864, Charcot described polyarthritis preceding pulmonary tuberculosis, and in 1892, Grocco proposed that arthritis could occur in patients with TB without direct bacillary invasion of the joints. In 1897, Poncet further characterized this entity as a sterile, non-erosive polyarthritis occurring during active TB, a condition later named Poncet's disease^12,13^.

Poncet's disease is currently recognized as a form of reactive arthritis triggered by TB, occurring without intra-articular infection and supporting the concept of a non-destructive, self-limited arthritis that resolves with TB treatment^14^. According to the diagnostic criteria proposed by Sharma and Pinto^15^, the diagnosis of Poncet's disease requires the presence of essential criteria—namely, inflammatory, non-erosive, non-deforming arthritis with exclusion of other causes of inflammatory arthritis—along with combinations of major and minor criteria. Our patient fulfilled these criteria, as he presented with non-destructive oligoarthritis and underwent extensive evaluation that excluded alternative causes of inflammatory arthritis. He also met both major criteria: microbiologically confirmed extra-articular tuberculosis and complete clinical remission of arthritis following antitubercular therapy. Among the minor criteria, the patient exhibited absence of sacroiliac and axial involvement. Therefore, this case meets the definition of definite Poncet's disease, which requires the essential criteria plus two major criteria.

Although synovial fluid analysis is traditionally performed to rule out infectious arthritis, its necessity in Poncet's disease remains debatable. In this case, the absence of clinical signs of joint effusion requiring drainage, lack of systemic sepsis, and rapid, complete response to antitubercular therapy argued against the need for invasive procedures. When there is strong clinical suspicion supported by evidence of systemic TB and symptom resolution with appropriate treatment, arthrocentesis may not provide additional diagnostic value and can reasonably be avoided—especially in pediatric patients, in whom the procedure carries additional risks.

Interestingly, our patient also presented with hypercalcemia in the context of suppressed PTH levels, most likely due to extrarenal calcitriol production by activated macrophages in granulomatous tissue, which is a well-documented but uncommon metabolic complication of disseminated TB^16^.

Remarkably, our patient fulfilled the SLICC classification criteria for cSLE, presenting with arthritis, serositis (pleural effusion), positive ANA, low complement levels (CH50), leukopenia, and lupus anticoagulant positivity^17^. Additional notable features included a high ANA titer (1:1280) and hypocomplementemia, both rarely reported in disseminated TB. This finding illustrates the limitations of classification criteria in immune-mediated rheumatic diseases, as infectious, malignant, and other inflammatory conditions may fulfill these criteria and lead to misdiagnosis. Moreover, TB and other secondary inflammatory or infectious processes may induce transient immunological abnormalities, including ANA positivity and less specific SLE-associated findings, such as complement consumption and antiphospholipid antibodies. Therefore, especially in TB-endemic settings, cSLE classification criteria should be applied only after careful exclusion of alternative diagnoses.

The decision to withhold immunosuppression was also crucial in this case. When cSLE is suspected, early initiation of corticosteroids is often routine. However, autoimmune rheumatic diseases are diagnoses of exclusion, and immunosuppressive therapy should be considered only after infectious and oncohematologic conditions have been definitively ruled out. In high-TB prevalence settings and in the context of diagnostic uncertainty, close monitoring and a careful stepwise investigation may prevent harmful treatment, particularly in pediatric patients with evolving features. The multidisciplinary approach in our tertiary care hospital, including input from pediatric infectious diseases, rheumatology, and immunology specialists, was essential to establishing the correct diagnosis.

We reinforce the recommendations of the World Health Organization (WHO) and the Brazilian Ministry of Health to perform HIV testing in all patients with a TB diagnosis^6,8^, since TB remains the leading cause of death among people living with HIV. In our patient, HIV testing was performed at the initial stage of the investigation due to the presence of fever, anemia, and lymphopenia.

## CONCLUSION

This case illustrates diagnostic challenges posed by overlapping features of disseminated TB and Poncet's disease mimicking cSLE. Recognition of Poncet's disease is essential to prevent misdiagnosis and potential harms of immunosuppressive therapy in patients with an underlying, treatable infection. In TB-endemic regions, clinicians must maintain a high level of clinical suspicion and include TB in the differential diagnosis of systemic inflammatory syndromes, especially in the presence of constitutional symptoms, cytopenias, and lymphadenopathy. Early identification and prompt initiation of TB treatment not only lead to clinical recovery but also prevent unnecessary interventions in these complex cases.

## Data Availability

The complete anonymized dataset supporting the findings of this study is included within the article.
